# Activity and Stability of Nanoconfined Alpha-Amylase in Mesoporous Silica

**DOI:** 10.1021/acsmaterialsau.3c00028

**Published:** 2023-08-04

**Authors:** Muhammad Naeem Iqbal, Aleksander Jaworski, Arthur C. Pinon, Tore Bengtsson, Niklas Hedin

**Affiliations:** † Department of Materials and Environmental Chemistry, Stockholm University, Stockholm SE-106 91, Sweden; ‡ Swedish NMR Center, University of Gothenburg, Gothenburg SE-405 30, Sweden; § Department of Molecular Biosciences, The Wenner-Gren Institute, Stockholm University, Stockholm SE-106 91, Sweden

**Keywords:** mesoporous silica particles, porcine pancreatic alpha-amylase, starch, 2-chloro-4-nitrophenyl alpha-d-maltotrioside (CNP-G3), (DNP) MAS NMR

## Abstract

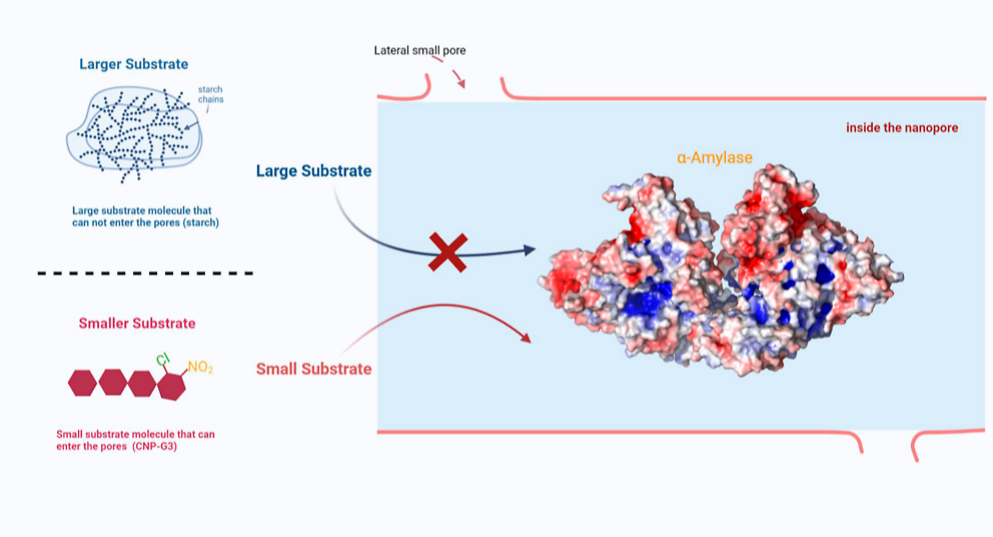

Mesoporous silica particles (MSPs) have been studied for their potential therapeutic uses in controlling obesity and diabetes. Previous studies have shown that the level of digestion of starch by α-amylase is considerably reduced in the presence of MSPs, and it has been shown to be caused by the adsorption of α-amylase by MSPs. In this study, we tested a hypothesis of enzymatic deactivation and measured the activity of α-amylase together with MSPs (SBA-15) using comparably small CNP-G3 (2-chloro-4-nitrophenyl alpha-d-maltotrioside) as a substrate. We showed that pore-incorporated α-amylase was active and displayed higher activity and stability compared to amylase in solution (the control). We attribute this to physical effects: the coadsorption of CNP-G3 on the MSPs and the relatively snug fit of the amylase in the pores. Biosorption in this article refers to the process of removal or adsorption of α-amylase from its solution phase into the same solution dispersed in, or adsorbed on, the MSPs. Large quantities of α-amylase were biosorbed (about 21% w/w) on the MSPs, and high values of the maximum reaction rate (*V*_max_) and the Michaelis–Menten constant (*K*_M_) were observed for the enzyme kinetics. These findings show that the reduced enzymatic activity for α-amylase on MSP observed here and in earlier studies was related to the large probe (starch) being too large to adsorb in the pores, and potato starch has indeed a hydrodynamic diameter much larger than the pore sizes of MSPs. Further insights into the interactions and environments of the α-amylase inside the MSPs were provided by ^1^H fast magic-angle spinning (MAS) nuclear magnetic resonance (NMR) and ^13^C/^15^N dynamic nuclear polarization MAS NMR experiments. It could be concluded that the overall fold and solvation of the α-amylase inside the MSPs were nearly identical to those in solution.

## Introduction

Mesoporous silica particles (MSPs) such as SBA-15 are characterized by a large surface area, variable mesopore size (2–50 nm), pore volume, mesocrystalline order, and ease of functionalization.^1,2^ These properties can be fine-tuned and are attractive for controlling molecular diffusion. The hydrodynamic dimensions of enzymes can be in the order of the pore size of MSPs, and nonobvious and diffusion-related effects may be expected.^3,4^ In addition to these features of diffusion and molecular control, this type of silica is researched for use in drug delivery, biocatalysis, and as therapeutic agents.^5–8^ Recent research has also explored the potential of MSPs for therapeutic application and large-scale production.^9,10^

Metabolic syndrome, which includes obesity and type 2 diabetes (T2D), is researched for its potential to be treated and prevented with MSPs. It was shown that amylase and lipase are adsorbed on MSPs in a pore-size-dependent manner both in vitro and ex vivo in gastric fluids. The pore-size dependency of the adsorption on MSPs is well-known,^11,12^ and it has been demonstrated that MSPs can act as a molecular sieve for proteins.^11,13^ Furthermore, it has been shown that MSPs pass through the gastrointestinal tract (GIT) of humans and mice without losing their structure and function.^10^ The apparent reduced activity of enzymes has been attributed to the size-specific adsorption of the enzymes (amylase and lipase) from complex gastric fluids and studied in vitro, ex vivo, and in vivo.^14,15^

The digestive enzyme α-amylase belongs to the glycoside hydrolase family and cleaves glucan links in polysaccharides, such as starch and glycogen. It is responsible for the release of maltose, etc. It has been shown that the activity of α-amylase from a variety of sources is drastically reduced when adsorbed on MSPs.^13,16,17^ Similar findings have been observed for other enzymes when adsorbed on MSPs.^18–21^ The reduction of enzymatic activity in the presence of MSPs can be of significance for the use of MSPs in drug administration and therapeutic and biocatalytic applications. This reduction could relate to the deactivation of the enzyme on adsorption, minor conformational changes, or complete loss of the secondary and tertiary structures that enzymes need to maintain for their function.^22–26^ However, the observed reduced activity could also relate to a substrate that is too large to fit the pores of the MSPs where the enzymes are presorbed.^27^ Particularly, the SBA-15 type MSPs, with tubular pores that enable the α-amylase to penetrate deep inside, ultimately limit the interaction with substrates with sizes larger than the pore diameters. In contrast, in mesoporous silica films (nanometer thick) with open surface porosity, the enzyme is located closer to the surface and can more readily interact with the substrate.^28^

In this study, we investigated the activity of porcine pancreatic α-amylase adsorbed on MSPs by using small (CNP-G3) and large (starch) probes. To gain further insight into the effects of the pore confinement, we conducted ^1^H/^13^C/^15^N magic-angle spinning (MAS) (DNP) nuclear magnetic resonance (NMR) experiments on the α-amylase biosorbed on the MSPs.

## Materials

CNP-G3 with product ID AB531477 was purchased from abcr Gute Chemie GmbH (Karlsruhe, Germany). 2-Chloro-4-nitrophenol (CNP) (cat no. C61208), porcine pancreatic α-amylase (cat no. A6255), 3,5-dinitrosalicylic acid (DNS—cat no. D0550), starch from potato (cat no. S2004), maltose (cat no. 1.05910), sodium chloride (cat no. S5881), potassium sodium tartrate tetrahydrate (cat no. S2377) and BCA assay kit (QuantiPro, cat no. QPBCA) were all purchased from (Merck, Germany). Phosphate buffer saline (PBS with 0.14 M NaCl, 0.0027 M KCl, and 0.010 M phosphate) (Medicago AB—Sweden) was adjusted to pH 5.4 with 0.1 M HCl and used for all the experiments with sorption and activity. Block copolymer (P123) and silica source tetraethylorthosilicate (TEOS) were purchased from Merck. Ethanol (98%), toluene, and HCl (37%) were all sourced from VWR (Sweden).

## Material Characterization

The MSPs were physically and structurally characterized by analyses of N_2_ sorption, low-angle X-ray diffraction (LAXRD), scanning electron microscopy (SEM), transmission electron microscopy (TEM), and NMR spectroscopy. Further details on the instrumentation and preparation procedures are presented in the Supporting Information.

### Biosorption Assay for Porcine Pancreas α-Amylase

The adsorption of porcine pancreatic α-amylase (A6255—Merck, Germany) on the mesoporous silica of SBA-15 type was studied using a colorimetric BCA (bicinchoninic acid) assay. All solutions were prepared in autoclaved Milli-Q grade water (MQ, from a Milli-Q system of Merk, Germany). These were sterilized before use by being passed through a 0.2 μm filter. Dispersions of silica were prepared in Milli-Q H_2_O by sonicating in a bath sonicator for 10 min at a final concentration level of 500 μg/mL to achieve a homogeneous dispersion. 60 μL of the dispersion was loaded in a 96-well polymerase chain reaction (PCR) plate. Porcine pancreas α-amylase suspensions were prepared from a stock of 0.37 mM concentration in the range of 0.09–1.85 μM in PBS at pH 5.4. 60 μL of pancreatic α-amylase solution of different concentrations was loaded on the silica dispersion holding plate. The plate was sealed and incubated at 37 °C for 3 h with vertical rotation (Harvard apparatus, cat no. 74-2302). Following incubation, the plate was centrifuged at 6200 G-force for 15 min to separate the MSPs from the supernatant. 60 μL of carefully drawn supernatant was transferred to a new flat-bottom plate (Corning, cat no. number 734-1657, VWR, Sweden) for determining the protein concentration by using BCA assay. The plate was incubated for another 60 min together with the BCA mixture in a 60 °C preheated oven. The plate was cooled, and measurements were performed at 562 nm using an absorbance reader (EnSpire, PerkinElmer, USA). All the absorbance values were blanked with a buffer. Finally, a standard curve was recorded, as shown in Figure S3, and fitted linearly. This fitting was used to calculate the final concentration of α-amylase. Successively, the quantity of α-amylase adsorbed by the silica (*Q*_e_) at equilibrium was determined as *Q*_e_ = (*C*_i_ – *C*_e_) *V*_tot_/*m* (silica). *C*_i_ is the initial concentration of α-amylase, *C*_e_ is the concentration of α-amylase at equilibrium, *V*_tot_ is the total volume, and *m* (silica) is the mass of silica. A nonlinear regression analysis that minimized the sum-squared deviations between the experimental data and a model for the specific amount of adsorbed protein (*Q*_e_) as a function of the protein concentration (*C*_e_) was performed. The analysis used the Hill model for adsorption and yielded parameters for the adsorption capacity (*m*_max_), interaction constant (*K*), and heterogeneity (*n*).
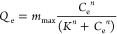
1

### Activity Assay for Porcine Pancreas α-Amylase within the Nanopores of Silica

A dispersion of MSPs (500 μg/mL) was prepared in MQ water under sonication, and an α-amylase solution (0.07 μM) in PBS (pH 5.4), each with a volume of 10 mL. These stocks were further used as such. Subsequently, an α-amylase solution (2.5 mL) was mixed with either a dispersion of MSPs or, as a control, in MQ water of equivalent volume. The final α-amylase solution (with or without MSP) had the same concentration of PBS (1×). It was kept for around 1 h, rotating in 5 mL sterilized vials, at 37 °C, in a temperature-controlled oven with vertical rotation (Harvard apparatus, cat no. 74-2302). This generated α-amylase loaded in MSPs and control (without loading, no silica). These were incubated at 37 °C either with starch (as a large probe molecule) or with 2-chloror-4-nitrophenyl alpha-d-maltotrisode (CNP-G3 as a small probe) in a 96-well plate, as described below. The final concentration of α-amylase in each well that contained substrate, α-amylase, and MSP (or control, no MSP) was about 0.02 μM. The reaction rate (*V*_0_) for the α-amylase was measured as described below and plotted with respect to change in the concentration of the substrates (starch or CNP-G3). The regression analysis used the Michaelis–Menten’s model (eq 2), and experimental and predicted data are shown in Figure 3b,c. In eq 2, [S] corresponds to the substrate concentration, *V*_max_ the maximum reaction rate, and *K*_M_ the Michaelis–Menten constant associated with the substrate binding affinity.
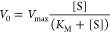
2

### Large Probe Molecule (Starch as Substrate)

Thirty μL of the MSPs loaded with α-amylase and the control sample with α-amylase without silica particles were incubated in the two separate flat-bottom PCR plates. These plates were preloaded with 30 μL of starch at concentrations ranging from 0.094 to 6000 μg/mL. A maltose solution in PBS was used as a standard. The plates having α-amylase (in MSP and free) and substrate were sealed and incubated at 37 °C for various lengths of time (15 min, 30 min, 1 h, 2 h, 3 h, 4 h, 5 h, 6 h, and 7 h). Following incubation, the plates were loaded with 60 μL of the 96 mM DNS solution and sealed with a high-temperature resistance seal. The plates were then kept at 96 °C for 15 min to allow the reduced sugars to react. Finally, the plates were cooled to room temperature and measured at 540 nm using an absorbance reader. All the absorbance values were blanked before being used for concentration measurement. The standard curve, which was linearly fitted, was used to estimate the amount of product (Figure S5).

### Small Probe Molecule (CNP-G3 as Substrate)

The same method used for starch was used for CNP-G3. We made the following adjustments. CNP-G3 (as a small probe substrate) was prepared with concentrations ranging from 0.06 to 4 mM, and CNP (in the same concentration range) was used as a standard. CNP-G3 (60 μL) loaded plates, which also held CNP standard in separate wells, were incubated with 40 μL of an α-amylase dispersion loaded in silica or α-amylase without silica (control). The plate was sealed and incubated at 37 °C with rotation for the indicated times. After predetermined intervals, the plate was recovered and the resulting CNP was evaluated at a wavelength of 405 nm. The PBS–MQ blank was used to create Figure S6, which displays the CNP standard curve. It was employed to calculate the yield from CNP-G3 digestion.

### DNP–NMR Characterization

The loading of α-amylase on the SBA-15 was achieved by dispersing the MSPs (500 μg/mL) in an α-amylase-containing solution (0.5 μM) and incubating the dispersion at 37 °C under slow rotation for 3 h. The mixture was spun down, and the supernatant was removed. At the end, the particles were rinsed once with the buffer. The MSPs with adsorbed α-amylase were lyophilized and stored partially dried in the buffer at a temperature of −20 °C until further use. The same procedure was used for the control sample (α-amylase with no SBA-15). ^1^H MAS NMR experiments were performed at a magnetic field of 14.1 T with a Bruker Avance-III spectrometer. The 1.3 mm probe head was used at a 60 kHz MAS rate. Acquisitions involved the use of a rotor-synchronized and double-adiabatic spin-echo sequence.^29,30^ Cooling with a BCU Extreme unit was employed to compensate for sample heating under fast MAS. DNP MAS NMR experiments were performed at a magnetic field of 9.4 T with a Bruker Avance Neo spectrometer, a 263 GHz gyrotron, and a 3.2 mm low-temperature probe head at a MAS rate of 12 kHz. The sample temperature was approximately 105 K. The magnetic field was set so that the microwave irradiation occurred at the same position as the positive-enhancement maximum for the AMUPol polarizing agent, which was used to enable the DNP enhancement of the signal-to-noise in the ^13^C and ^15^N NMR spectra. The DNP-enhanced cross-polarization (CP) ^13^C and ^15^N MAS experiments employed a SPINAL-64 heteronuclear decoupling scheme, whereas the DNP-enhanced ^1^H–^13^C CP-HETCOR correlation experiment employed homonuclear ^1^H decoupling of the frequency-switched Lee–Goldburg (FSLG) type. See (Supporting Information) for more details.

## Results and Discussion

The MSPs (SBA-15) were prepared with sufficiently large pores (11.2 nm) to allow a significant amount of α-amylase adsorption. The MSPs were studied with respect to the α-amylase adsorption and enzymatic activity using a large (potato starch) and a small (CNP-G3) probe (substrate). The MSPs were also studied physically and structurally using N_2_ gas sorption analysis, LAXRD, SEM and TEM, and 2D HETCOR DNP–NMR spectroscopy.

### Physical and Structural Characterization of the MSPs

**Figure 1:**
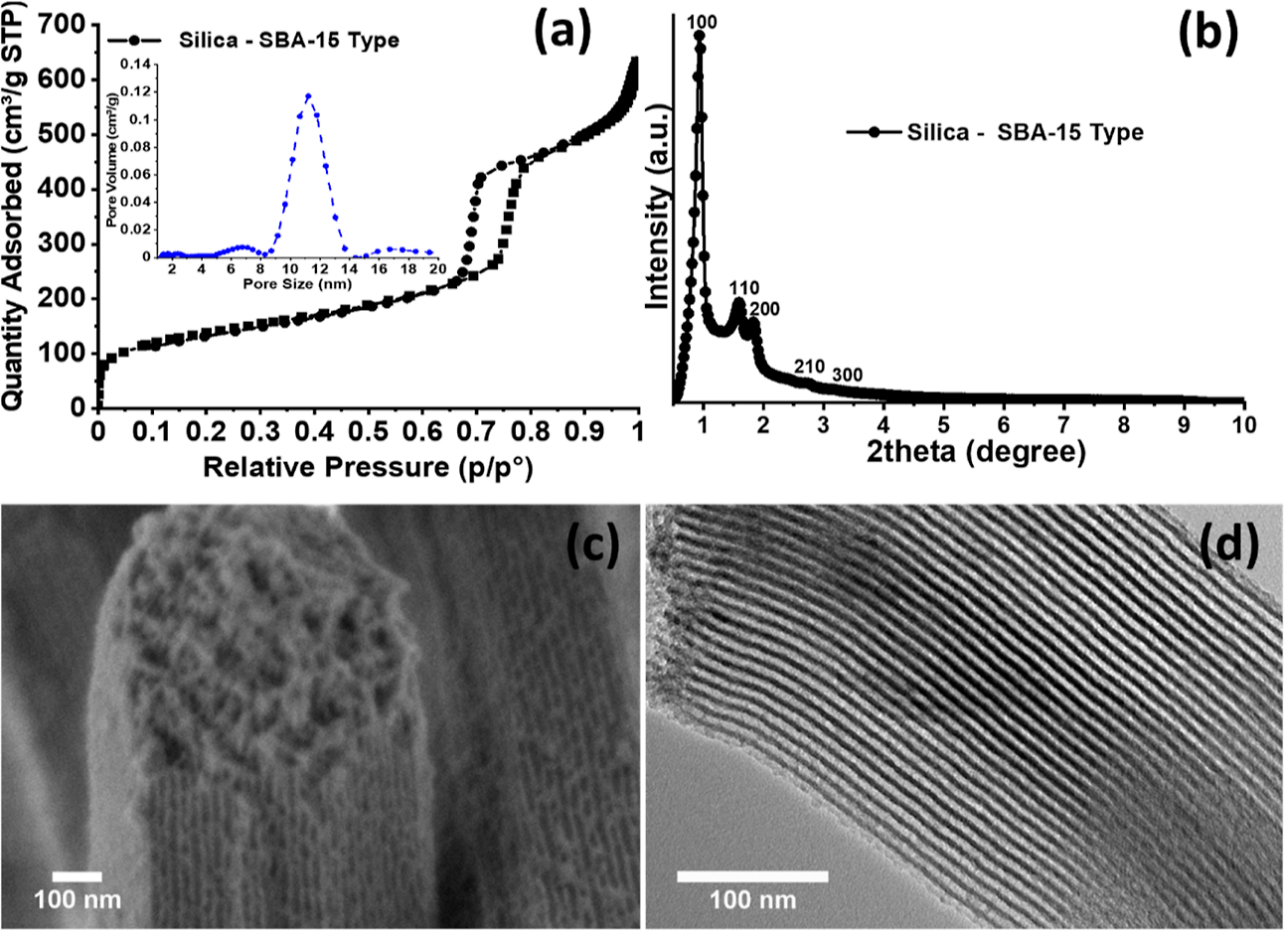
Physical characteristics of the mesoporous silica of SBA-15 type used for adsorption of α-amylase and related activity testing. (a) N_2_ gas adsorption–desorption isotherms with an inset plot of the pore size distribution, (b) LAXRD of the particles, (c) an SEM image of the curved rod-like particles, and (d) a TEM image of the porous network in a particle.

N_2_ sorption isotherms for the calcined MSPs were recorded to allow for an analysis of the pore size distribution. The corresponding N_2_ adsorption and desorption isotherms are presented in Figure 1a. The MSPs had a typical type IV isotherm^31^ with an H1-type hysteresis loop,^32^ and a pore size distribution centered on 11.2 nm, having a full-width half maximum (fwhm) of 2.5 nm. The specific surface area (BET—Brunauer–Emmett–Teller)^33^ was 518 m^2^/g, and the micropore surface area (*t*-plot) 131 m^2^/g. The total pore volume for the studied MSPs was 1.03 cm^3^/g. The structural ordering of the MSPs^34^ was studied with LAXRD analysis,^35,36^ the results of which are presented in Figure 1b. The pattern was typical of a mesoporous ordered hexagonal structure with well-resolved reflections at (100), (110), and (200), and two additional peaks (210) and (300) were also present. The high-resolution SEM image in Figure 1c shows that the particles were rod-shaped and had a length of approximately 2 μm and a diameter of 0.5 μm. This size and mesostructured ordering were further confirmed by analysis of HRTEM images such as that in Figure 1d. The particles were relatively uniformly sized, and the long pore channels are visible in the HRTEM image.

### Biosorption of α-Amylase

**Figure 2:**
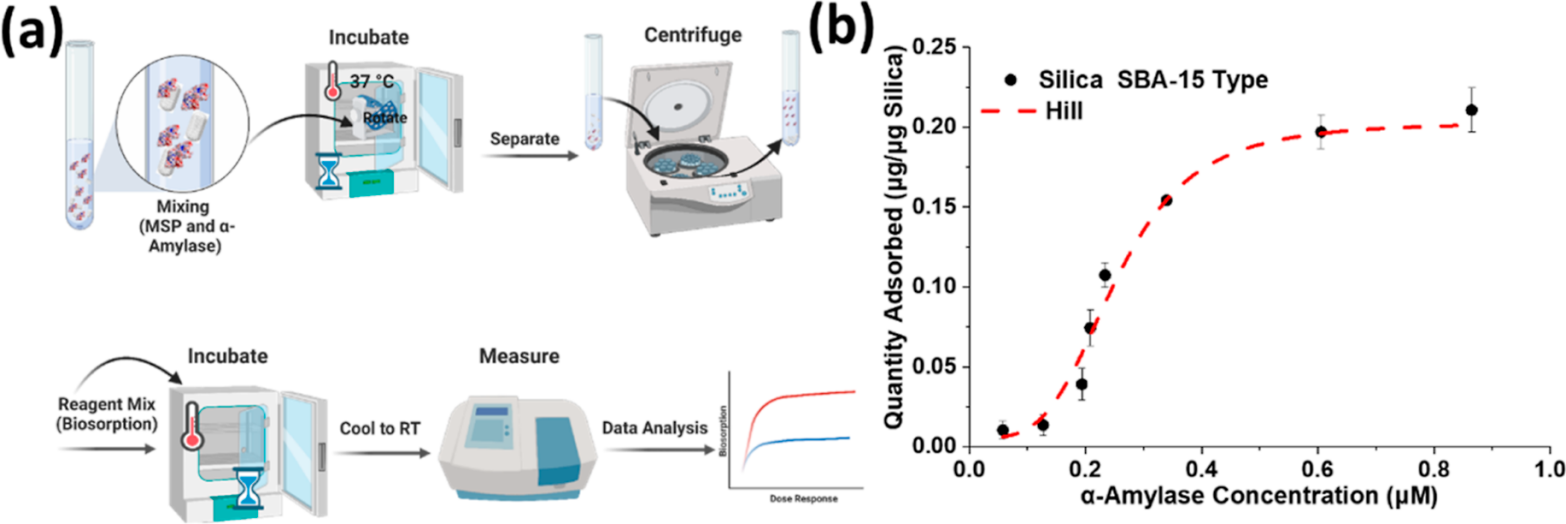
(a) General scheme used for measuring (b) the biosorption of α-amylase in mesoporous silica particles of the SBA-15 type. The values are the mean and SD of triplicate experiments.

The adsorption of α-amylase on the MSPs was studied following an established approach,^14^ and the overall layout is shown in Figure 2a. The dose-dependent adsorption of α-amylase had a sigmoidal dependency, as shown in Figure 2b. The adsorption was analyzed in the Hill model (eq 1),^37^ and the *K* was 0.25 ± 0.02 μM. The *m*_max_ was 21% (w/w) and corresponded to a pore filling of about 56% (w/w), which was slightly higher than that in a previous study.^14^ A thermal gravimetric analysis consistently revealed that α-amylase biosorbed in the silica was around 24%, as estimated from the mass within the temperature range of 200–600 °C; the corresponding gravimetric traces are presented in Figure S8 (Supporting Information). The postsynthesis treatment applied to the MSPs led to an unplugging effect, which in turn generated more open pore entrances and the high increase in adsorptive capacity. It is well established that the adsorption of enzymes in rod-like SBA-15-type particles can be enhanced by controlling mass-transport restrictions.^38^ Measured parameters for the α-amylase adsorption and textural properties after postsynthesis treatment of the MSPs are presented in Tables S1 and S2. It can be noted that the MSPs studied were of the SBA-15 type and had mesopores with an average diameter of 11.2 nm to accommodate well the porcine-pancreatic α-amylase. Alpha-amylase has a hydrodynamic diameter of 7–8 nm. In another study, experiments were conducted with similar SBA-15 particles, and the adsorption of α-amylase on particles with closed pores (polymer-filled) and empty pores (calcined particles) were compared. It was observed that α-amylase was adsorbed exclusively in the empty pores and not on the outer surface of the particles.^14^

### Nanoentrapment Effect on the Activity of Porcine Pancreas α-Amylase

**Figure 3:**
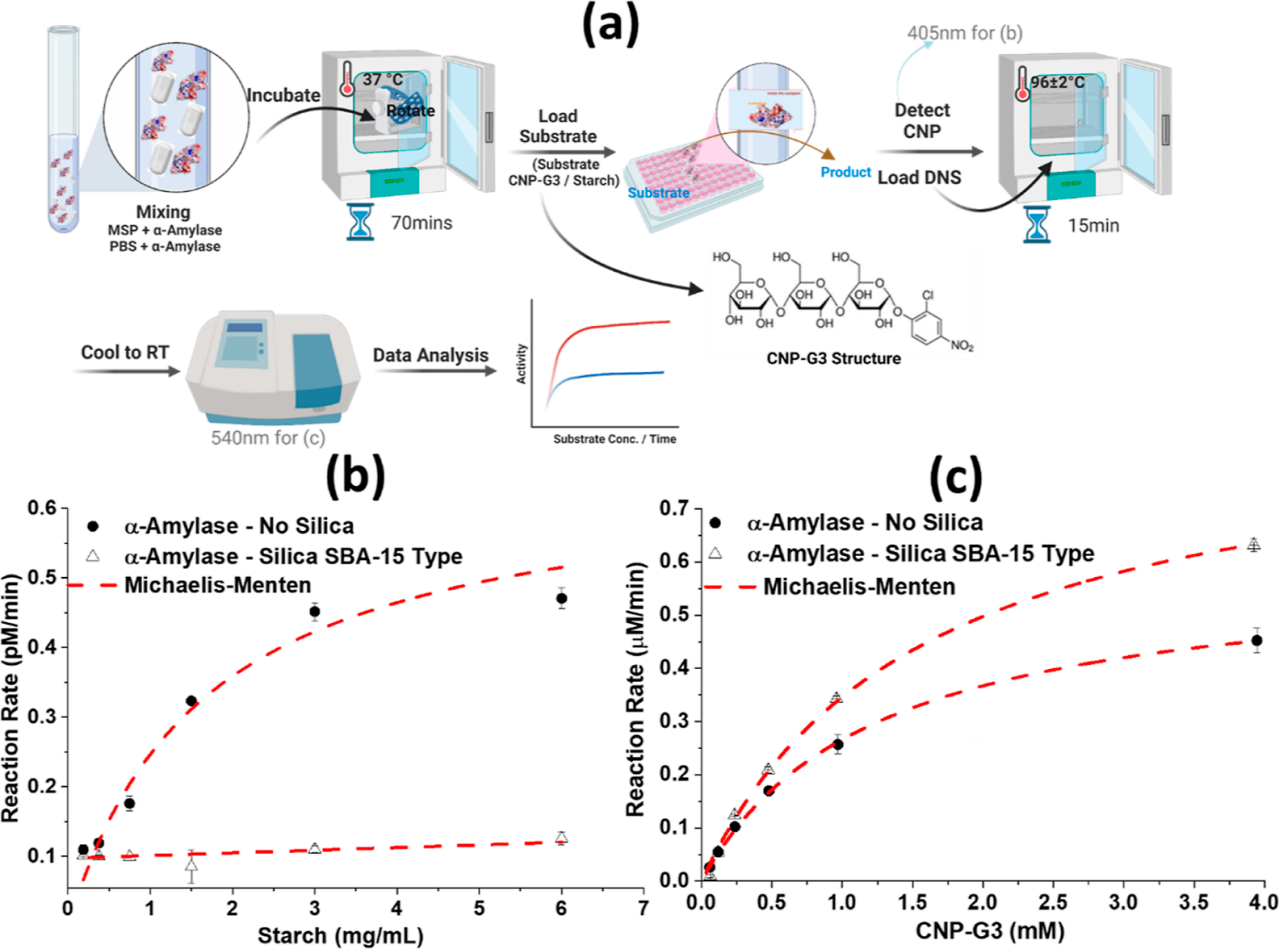
(a) General scheme used for measuring the activity with the (b) large probe/substrate (soluble starch) as a substrate with variable concentration and (c) small probe/substrate (CNP-G3) with and without the nanoentrapment of α-amylase inside the nanosized channels of SBA-15. The concentration of α-amylase in each case remained the same (0.02 μM). Values are the mean and SD of triplicate experiments.

The activity of α-amylase adsorbed on MSPs was studied with large and small probe molecules (substrates), and the overall approach is illustrated in Figure 3a.

### Large Probe Molecule—Starch

The activity of α-amylase adsorbed on MSPs was very low when using the large probe molecule (starch). The reaction rate was determined for several concentrations of starch, as can be seen in Figure 3b. The potato starch used in this study had a typical molecular weight of about 4.26 × 10^6^ g/mol and a hydrodynamic radius of >50 nm (Figure S7).^39^ This hydrodynamic radius is significantly larger than the average pore diameter of the MSPs, which was 11.2 nm. The activity for the control sample (dissolved α-amylase) was high. This suggested that starch was too large to adsorb within the pores of the MSPs. The experimental setup for this experiment was purposely chosen to mimic the in vivo and clinical conditions inside the GIT of humans and animals and the general scheme for the setup is shown in Figure 2a. This setup was chosen over one that included a physical separation of the MSPs and the α-amylase solution, followed by redispersion in a fresh buffer for activity measurement, which has typically been used for this type of enzyme activity studies.^40^

The time dependency of the specific activity decreased in the case of both the control and the α-amylase adsorbed on MSPs, as can be seen in Figure S2b. The specific activity stabilized after 4 or 5 h. There was a small static activity for the α-amylase adsorbed on MSPs with a reaction rate of 0.1 pM/min. This finding suggests that the adsorbed enzyme did not leach over time and reinitiated the digestion of starch. The initially higher activity for the enzyme adsorbed on the MSPs, compared with the control, was attributed to enzymes adsorbed at the pore entrances or on the outer surface of the MSPs, or from a small remaining fraction in the liquid phase.^15^ Our results that α-amylase adsorbed on SBA-15 was not active toward starch might appear to stand in contrast to the study of Bellino et al.^28^ They studied nanometer-sized mesoporous films with immobilized α-amylase that were active toward starch. We explain the difference between their and our study by highlighting that the porous and nanometer-thick films (85 and 135 nm) in their study fall within the size limits of the starch substrate. It was also noted that their films had a more open porosity than the particles in this current study.

### Small Probe Molecule—CNP-G3

The activity of α-amylase adsorbed on MSPs was high when determined with the small probe molecule (CNP-G3) and higher than that for the control (free α-amylase), as can be seen from the reaction rates in Figure 3c. CNP-G3 has three glucose units bonded through α-glycosidic bonds and CNP as an indicator. CNP-G3 has an estimated hydrodynamic radius of <1 nm (Figure S7), which is smaller than the pore diameter of the MSPs (11.2 nm). The Michaelis–Menten equation^41,42^ was employed for the analysis of the data from the activity measurements. The corresponding *K*_M_ and *V*_max_ are listed in Table 1. The increased *K*_M_ and *V*_max_ for α-amylase adsorbed on MSPs confirmed that the biosorbed enzyme was more active than the free enzyme (control). Furthermore, the adsorbed enzyme was found to be more stable than the free one. In a typical enzymatic inhibition, one or both of the *K*_M_ and *V*_max_ groups tend to decrease. On the contrary, shifts of both *K*_M_ and *V*_max_ have been observed toward high values with molecular crowding agents (such as dextran, polyethylene glycol, etc.).^43–45^ A related molecular crowding was expected by the biosorption on MSPs, and slightly higher values of *K*_M_ and *V*_max_ were indeed observed as compared with the control. The specific activity did not decrease as quickly for the α-amylase adsorbed on MSPs as compared to that of the free enzyme, as shown in Figure S2a. Mass-transport limitations were observed in relation to the activities of α-amylase adsorbed in the pores of MSPs. We speculate that the effect of coadsorption of the CNP-G3 within the nanopores may be in part responsible for the enhanced activity, and other contributions could be in the stabilization of the enzyme on adsorption. It was mass-transport limitations. We here presumed that the heat of adsorption of CNP-G3 was not higher than that of the amylase during adsorption in the SBA-15 particles. Previous studies have demonstrated that α-amylase effectively fits within the nanochannels of large-pore SBA-15 silica,^46^ suggesting that it can undergo rotational diffusion in the pores due to the size matching and features of the hydration layer on the surface of the silica pores. This type of motion is known as a solvent-slaved motion and has been reported to play an important role in the function of certain biomolecules.^47,48^

**Table 1:** Parameters for the Michaelis–Menten Model (eq 2) for the Free and Adsorbed Enzyme^a^

	small probe: CNP-G3 as substrate		large probe: starch as substrate	
sample	α-amylase—no silica	α-amylase—silica SBA-15 type	α-amylase—no silica	α-amylase—silica SBA-15 type
description	free α-amylase	nanoentraped α-amylase	free α-amylase	nanoentraped α-amylase
*V*_max_	0.592 ± 0.024 μM/min	0.885 ± 0.05 μM/min	0.659 ± 0.076 pM/min	N/A
*K*_M_	1.221 ± 0.117 mM	1.555 ± 0.202 mM	1.673 ± 0.276 mg/mL	N/A
*R*2	99.6%	99.4%	97.4%	N/A

a CNP-G3 (small probe) and starch (large probe) were used as substrates.

### Dynamic Nuclear Polarization MAS NMR Characterization

**Figure 4:**
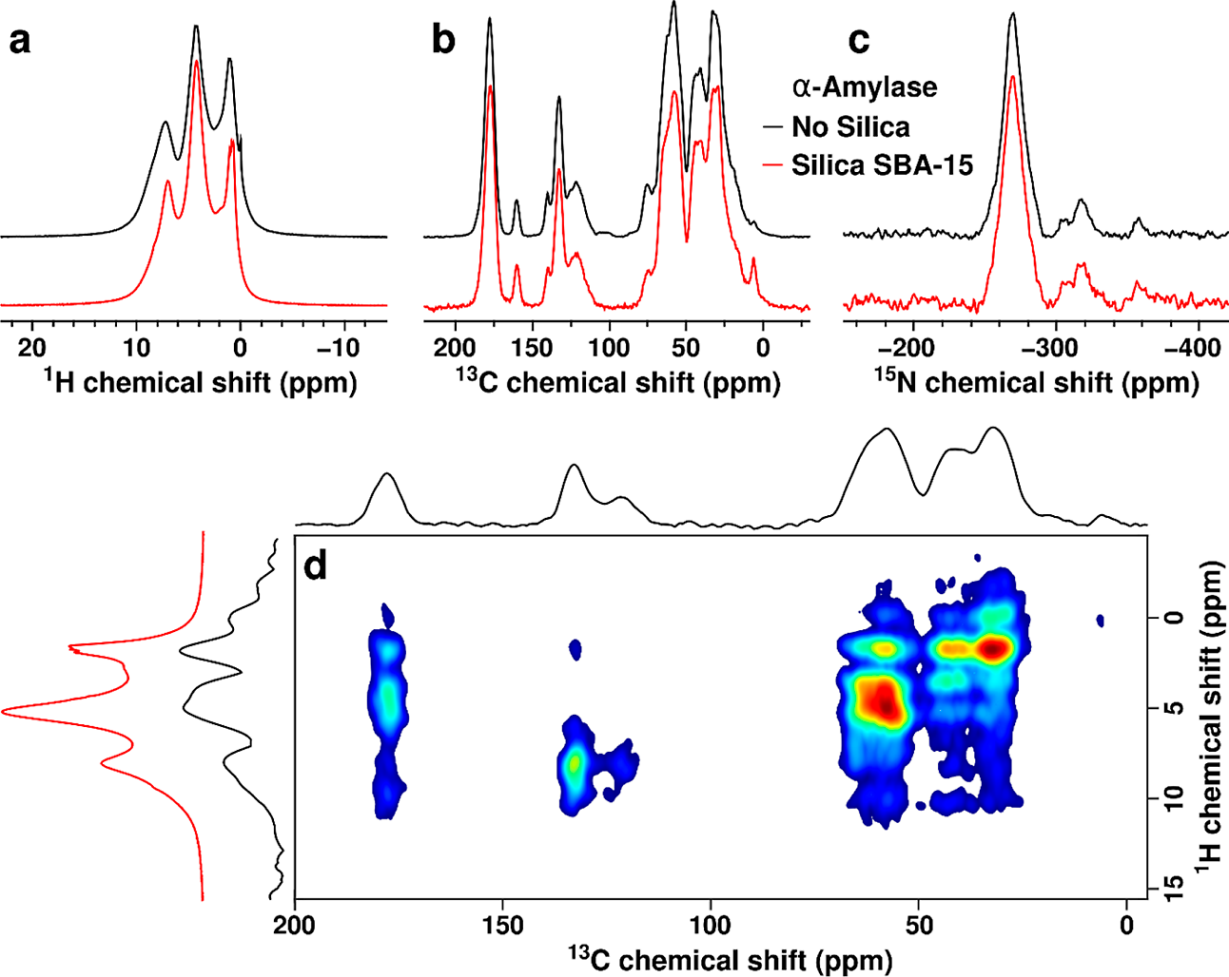
(a) ^1^H MAS (60 kHz) and (b) ^13^C and (c) ^15^N DNP MAS (12 kHz) NMR spectra of α-amylase without silica (no silica; black traces) and α-amylase biosorbed inside silica (silica-SBA-15; red traces). (d) A DNP–FSLG–HETCOR ^1^H–^13^C NMR correlation spectrum of α-amylase biosorbed inside the silica is shown together with projections in both dimensions. The projection in the indirect (^1^H) dimension (black trace) is compared to the corresponding fast MAS ^1^H NMR spectrum (red trace).

To gain further insights into the potential solvation and conformational changes of biosorbed α-amylase, different types of NMR experiments were conducted. From preliminary MAS NMR experiments performed on α-amylase adsorbed in SBA-15, it was concluded that the signal-to-noise ratio in the spectra was not high enough to determine if the α-amylase was folded in the adsorbed state without the use of DNP experiments. For the detailed analysis, ^1^H NMR spectra were recorded under the conditions of fast MAS, and DNP-enhanced ^13^C and ^15^N NMR spectra were recorded at low-temperature and under MAS. The DNP was applied to increase the ^13^C and ^15^N NMR sensitivity at natural isotope abundance.^49^ We used AMUPol as a polarizing agent, which is recognized as appropriate for use in biological systems according to recent literature.^50,51^ The ^1^H NMR spectra of the free (control) and biosorbed α-amylases are presented in Figure 4a and have no significant differences. The signals at ^1^H NMR chemical shifts of 0–2 ppm belong to aliphatic groups, the one at around ∼4.7 ppm to water, and the one at ∼7 ppm to aromatic groups. Importantly, the intensity of the H_2_O signal was only slightly increased for α-amylase biosorbed on the MSPs compared to the control (pure α-amylase). This similarity indicates that the solvation of α-amylase is similar, despite the large internal volume of the MSPs. We used the similarities of the ^13^C and ^15^N NMR spectra for adsorbed α-amylase and free α-amylase to conclude that the adsorbed amylase was folded. The DNP-enhanced ^13^C NMR spectra in Figure 4b have relatively narrow bands, which are typical for folded proteins. The ^13^C NMR chemical shifts were assigned to side-chain aliphatic carbon atoms (15–45 ppm) and Cα atoms (45–70 ppm); side-chain aromatics (110–140 ppm), arginine Cζ and tyrosine C4 (160 ppm), and C=O groups (175–180 ppm).^52^ The DNP-enhanced ^15^N NMR spectra shown in Figure 4c reveal typical backbone amide signals with ^15^N chemical shifts between −260 and −280 ppm as well distinct resonances of Nε and Nη atoms of arginine at around −305 and −315 ppm, respectively, and that from the NH_3_^+^ group of lysine at −355 ppm.^52^ The DNP-enhanced ^13^C and ^15^N NMR spectra of the free and biosorbed α-amylase are essentially identical; hence, no conformational changes of the enzyme structures are expected to have occurred on the adsorption of the α-amylase on the MSPs. The similarities of the ^13^C and ^15^N NMR spectra for adsorbed α-amylase and free α-amylase were used to conclude that adsorbed α-amylase was folded. In addition to the ^1^H NMR spectrum recorded at a high temperature (Figure 4a), a related ^1^H NMR spectrum was recorded at a low temperature (105 K) for α-amylase adsorbed on the MSPs using the projection of a ^1^H homonuclear decoupled 2D DNP–FSLG–HETCOR NMR spectrum. It is presented in Figure 4d. Here, it is worth noting that ^13^C{^1^H} HETCOR NMR spectra of this kind have been used without DNP. For example, Sardo et al. used this technique to study the details of the chemisorption of CO_2_ on aminated silica.^53^ However, to the best of our knowledge, this approach to achieving high resolution in the ^1^H projection spectrum has not been documented before in the open literature for DNP-enhanced NMR and porous silica systems. As can be observed by comparing the red and black traces in Figure 4d, the ^1^H (*t*_1_) projection of the 2D DNP–FSLG–HETCOR NMR spectrum (black trace) has a shape that compares well to that of the fast-MAS ^1^H NMR spectrum (red trace). In this way, we demonstrate that it is possible to obtain high-resolution ^1^H NMR spectra under MAS (at 12 kHz) and low temperatures (105 K), avoiding elevated temperatures associated with fast MAS. This avoidance could be crucial for certain sensitive biological systems that might undergo conformational changes and partially/completely unfold under the conditions of fast MAS.^54,55^ Note that additional signals that do not belong to α-amylase are discernible in the DNP-enhanced spectra: those from the cryoprotectant (glycerol, ^1^H shifts ∼4 ppm) and the silicon polymer plug of the DNP rotor (^1^H/^13^C signals at 0/∼10 ppm).

**Scheme 1:**
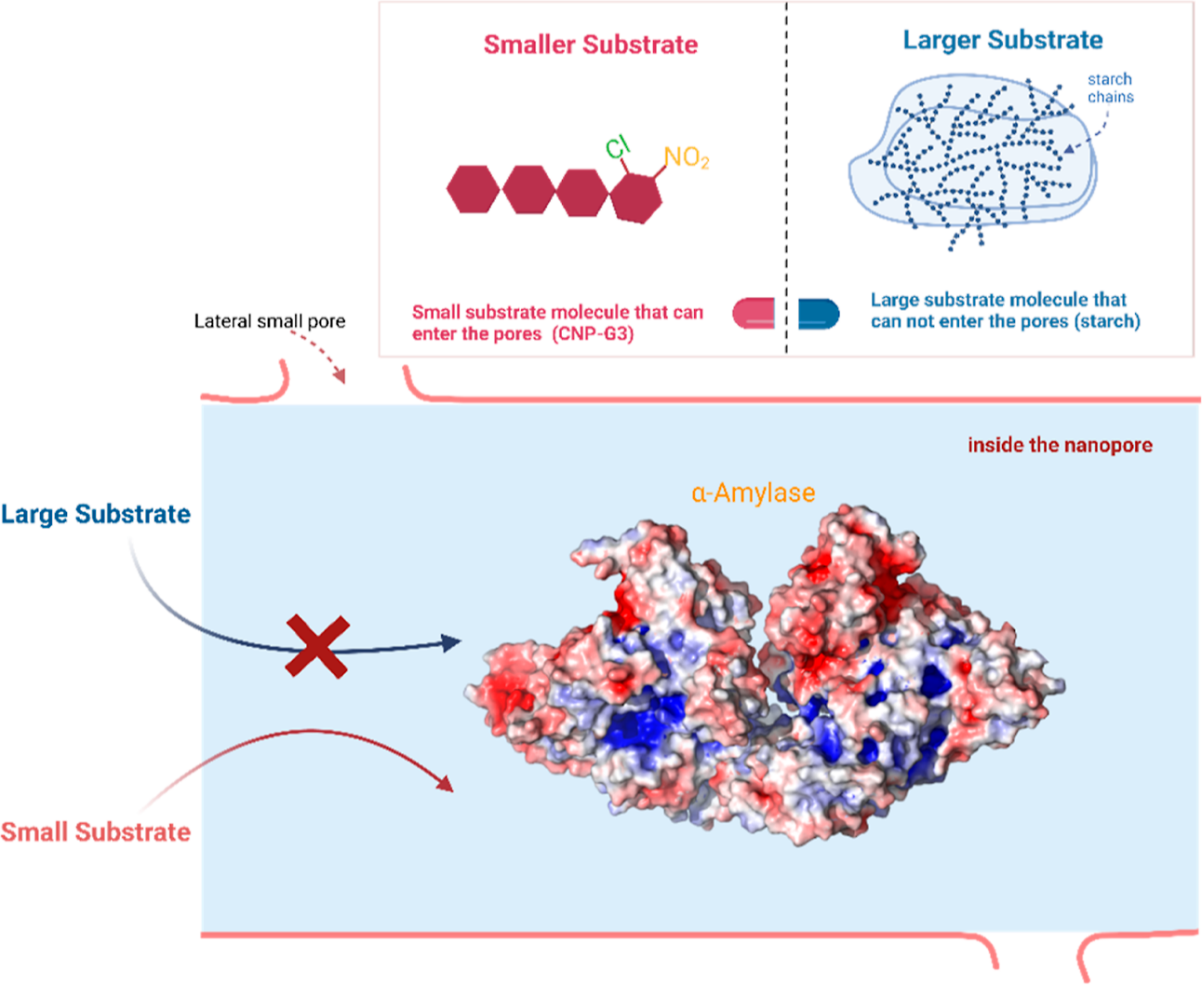
Possible Positioning of the Biosorbed α-Amylase within the Nanopore of the SBA-Type Silica The large probe/substrate is starch, which cannot enter the pore because of size restriction, whereas the smaller probe/substrate CNP-G3 (2-chloro-4-nitrophenyl-α-d-maltotrioside) can easily access adsorbed α-amylase.

From the high enzymatic activity observed with the small CNP-G3/MSPs probe and NMR analysis, it is clear that α-amylase adsorbed on MSPs is active, as illustrated in Scheme 1. The enzyme is not denatured in the pores of the MSPs, which could relate to the fact that the folded and native state of the enzyme has its hydrophobic part buried within its structure. Also, silica has a hydration layer, approximately 1–2 nm thick,^46^ which may provide assistance to the local movement of adsorbed α-amylase and allow access to the active site via rotational diffusion of the enzyme within solvent-filled pores marginally larger than the hydrodynamic diameter of the enzyme. This tight confinement could have helped to inhibit unfolding because such a process would require an increase in the molecular volume of α-amylase.^56,57^ Thus, the enhanced long-term activity observed for biosorbed α-amylase, as compared to the control in solution, supports the possibility that such a stabilization could have been active.^58–60^

## Conclusions

Pancreatic α-amylase adsorbed in the pores of MSPs displayed high activity and retained its conformation. The pore size of SBA-15-type silica was 11.2 nm, which was larger than the hydrodynamic dimension of the enzyme. The adsorption capacity was about 21% (w/w). When the small probe molecule was tested (CNP-G3), the activity was high. No activity was observed with the large probe molecule (potato starch). The latter had a hydrodynamic diameter much larger than the pore size of the silica. The Michaelis–Menten parameters *K*_M_ and *V*_max_ were comparable for the adsorbed α-amylase and the control (in solution). These observations suggest that the substrate affinity and catalytic efficiency were not significantly affected by confinement within the pore. However, it is worth noting that a higher activity over long periods was observed specifically for the smaller substrate (CNP-G3) for the adsorbed α-amylase than for the control sample, which indicated an enhanced stabilization of the enzyme within the nanopores.

The MAS ^1^H NMR and ^13^C, ^15^N DNP MAS NMR spectra of the pure (free) and adsorbed α-amylase in the MSPs were essentially identical, suggesting that the overall conformation and solvation of the enzyme were similar.

With our hypothesis regarding the importance of a size match between the probe/substrate and the pores of the MSPs in mind, it can be highlighted that one should not directly conclude that a protein has been deactivated solely based on a recorded absence of enzymatic activity. This situation was demonstrated for α-amylase adsorbed on MSPs, and we expect that it can be applied to a range of other enzymes as well. It should be noted that the pore diameters of SBA-15 and other MSPs can be tuned over a wide range of sizes. This study also showed that high-signal-to-noise DNP–FSLG–HETCOR MAS ^1^H–^13^C MAS NMR and ^15^N DNP MAS NMR spectra could be recorded for unlabeled α-amylase adsorbed on MSPs. Obtaining similar spectra should be feasible for related adsorbed or otherwise diluted systems of proteins in the solid state.

The use of the small probe molecule CNP-G3 as a substrate enabled us to demonstrate high activity for α-amylase adsorbed in the MSPs. This activity was found to be more extended in time for adsorbed α-amylase than the control (enzyme in a buffer), which is a new finding, as related studies have focused on the reduced activity of a large probe molecule (starch) too large to adsorb in the MSPs. Our results also confirm an earlier hypothesis that biomolecules are physically separated from the GIT of mice and humans when MSPs are orally ingested. Ingestion seems to hinder the interaction of such biomolecules with large molecules and structures related to the food ingested, such as starch.^14^ Additionally, we emphasize one finding concerning the solid-state DNP NMR methodology used. The indirect FSLG decoupling enabled a resolution in the ^1^H NMR domain that was much higher than what would have been achieved with a more standard implementation of the DNP HETCOR NMR experiment.

The findings from this work should be relevant to a variety of systems with immobilized enzymes for biocatalysis and other purposes. The techniques of selecting suitable probe molecules and conducting solid-state NMR experiments using DNP should both be relevant for studies of a broad range of enzymatic reactions in confined spaces.

## Supporting Information Available

Material characterization techniques, NMR experimentation, post-synthesis treatment of particles, characterization data (LAXRD and porosity) on preprocessed MSPs, biosorption analysis parameters for MSPs after each processing step, porosity data of MSP after each processing step, specific activity over a long time period, a standard curve for α-amylase concentration measurement, standard curves for product concentration measurements, and size analysis of substrates (CNP-G3 and starch) (PDF)
