# Supplementary material for: Activity and Stability of Nanoconfined Alpha-Amylase in Mesoporous Silica
Source: ACS Mater Au. 2023 Aug 4;3(6):659–68. doi: 10.1021/acsmaterialsau.3c00028 (PMC10636787; doi:10.1021/acsmaterialsau.3c00028)
Supplement: Supplementary file 1 — mg3c00028_si_001.pdf [file mg3c00028_si_001.pdf]

Supporting Information for

Activity and Stability of Nano-confined alpha-Amylase in Mesoporous Silica

Muhammad Naeem Iqbal[1], Aleksander Jaworski[1], Arthur C. Pinon[2], Tore Bengtsson[2], Niklas Hedin[1*]

[1] Department of Materials and Environmental Chemistry, Stockholm University, Stockholm, SE-106 91, Sweden

[2] Swedish NMR Center, University of Gothenburg, Gothenburg, SE-405 30, Sweden

[2] Department of Molecular Biosciences, The Wenner-Gren institute, Stockholm University, Stockholm, SE-106 91, Sweden

[*] Corresponding Authors

[niklas.hedin@mmk.su.se]

Number of Pages: 14

Number of Figures: 8

Number of Tables: 2

Materials Characterization

Nitrogen sorption analysis

N_2 sorption analysis was performed with a Tristar II instrument (Micromeritics, USA) at the temperature of liquid N_2. All the samples from Scheme SI-1 were subjected to dynamic vacuum conditions and a temperature of 150°C for at least 19 hours before being studied with N_2 gas sorption. The specific surface area was determined in the BET model using adsorption data in the relative pressure regime of 0.05–0.20. The total pore volume was determined by single point adsorption at a relative pressure close to unity. The micropore surface area and pore volume were determined by the t-plot method within the thickness range of 0.5–0.8 nm. Density functional theory (DFT) was applied to the N_2 adsorption data using a cylindrical pore model for pore size distribution (PSD) estimation, [1] and a regularization procedure was used to avoid over interpretation of the data, using a program from Micromeritics (USA). All the measurements were at least triplicated and presented as average with errors as standard deviation.

DNP-NMR Characterization

The loading of α-amylase on the SBA-15 was achieved by dispersing the MSP (500µg/mL) in an α-amylase solution (0.05 nM) and incubating the dispersion at 37°C under slow rotation for 3 hours. The mixture was spun down and the supernatant was removed, at the end particles were rinsed once with the buffer. The MSP with adsorbed α-amylase was lyophilized and stored partially dried in the buffer at a temperature of -20°C until further use. The same procedure was used for the control sample (α-amylase with no SBA-15). ^{1}H magic-angle-spinning (MAS) NMR experiments were performed at a magnetic field of 14.1 T (Larmor frequency of 600.12 MHz) on a wide-bore Bruker Avance-III spectrometer. The 1.3 mm probe head was used with a 60 kHz MAS rate. Acquisitions involved use of a rotor-synchronized,

double-adiabatic spin-echo sequence with a 90° excitation pulse of 1.25 μs followed by a pair of 50.0 μs tanh/tan short high-power adiabatic pulses (SHAPs) with 5 MHz frequency sweep., All pulses operated at the nutation frequency of 200 kHz, and 128 signal transients were acquired using a relaxation delay of 5 s. Cooling with a BCU Extreme unit was employed to compensate for sample heating under fast MAS. DNP solid-state NMR experiments were performed on a wide bore 9.4 T Bruker magnet (Larmor frequencies of 400.27, 100.64, and 40.56 MHz for ^{1}H, ^{13}C, and ^{15}N respectively) with an Avance Neo spectrometer, a 263 GHz gyrotron, a low temperature cooling cabinet, and a triple resonance 3.2 mm low-temperature probe in the ^{1}H/^{13}C/^{15}N mode. The sample was spinning at 12 kHz MAS frequency. The sample temperature for the DNP experiments was approximately 105 K. The field sweep coil of the main magnetic fields was set so that microwave irradiation occurred at the same position as the positive enhancement maximum for AMUPol polarizing agent. Cross-Polarization (CP) MAS NMR experiments were performed with a contact pulse on ^{1}H which was linearly ramped from ν_1 = 100 to 110 kHz and from 50 to 55 kHz for ^{13}C and ^{15}N experiments, respectively; ^{13}C and ^{15}N CP spin lock RF field amplitudes of 65 and 25 kHz were used, respectively. The SPINAL-64 heteronuclear decoupling scheme was applied during acquisition with ^{1}H RF fields of ca. 100 kHz. Between 2048 and 28672 signal transients were recorded with a recycling time of 2 s. The contact times used for the CP spin lock were found to be optimal between 500 and 1750 μs. The DNP-enhanced ^{1}H-^{13}C CP-HETCOR correlation spectrum employed the Frequency Switched Lee-Goldburg (FSLG) homonuclear ^{1}H decoupling during t_1 evolution, which was applied at the RF field strength of approximately 100 kHz. ^{1}H and ^{13}C chemical shifts are reported with respect to t*etramethylsilane* (TMS), and ^{15}N chemical shifts w.r.t. nitromethane (CH_3NO_2).

Low-angle X-ray diffraction (XRD)

Low-angle XRD was performed on Bruker D8 Discovery powder diffractometer operated at 40 kV and 40 mA, with 2min/° and equipped with Cu Kα radiation source. in a grazing incidence X-ray Diffraction. The grazing incident X-Ray diffraction techniques with very small incident angle beam to the sample surface was used. Powdered samples were prepared in an amorphous quartz holder by filling approximately 1 mm depth. The unit cell (**a_0**) parameter was calculated using $(2/\sqrt{3})$ d_{100} from the d_{100} spacing, assuming a 2D hexagonal pore geometry.

Scanning electron microscopy (SEM)

Morphological investigations of the nanoporous silica of SBA-15 type samples taken at every stage according to Scheme SI-1 were performed with SEM. Images were recorded with a JSM 7401 (JEOL, Japan) microscope using a secondary electron detector. It is equipped with cold field emission gun as an electron source. The microscope was typically operated at an accelerating voltage of 0.3kV-1kV, stage bias of up to 2.75 kV and probe current of several Pico amperes was sometimes also used. The working distance was altered between 2 and 3 millimetres. Samples were prepared by dispersing a small amount of particles in dichloromethane by ultrasonication for 5 minutes, depositing one drop of the dispersion on a pre-ground aluminum substrate and letting to dry before loading in the microscope. Images were taken on non-coated samples.

Transmission electron microscope (TEM)

Transmission electron microscope analysis was performed with a JEM-2100F (JEOL, Japan) microscope, equipped with LAB6 as electron-source filament, for visualizing the porous network of the nanopoour silica samples. It was operated at 200 kV and imaged using a bottom mounted CCD (Gatan SC1000 ORIUS) camera. Samples for

TEM imaging examination were prepared by sonicating the pre weighed dried particles at 250µg/mL concentration in pure ethanol for 10 min. A 10µL of this particle dispersion was then transferred to a carbon-coated copper grid.

Biosorption analysis using α-amylase from porcine pancreas

The protein adsorption experiments used α-amylase from porcine pancreas (A6255 – Sigma Aldrich, Merck). The amount of α-amylase was measured using a bicinchoninic acid (BCA) assay kit (QuantiPro, Sigma-Aldrich, USA, cat no. QPBCA). Measurements of absorbances were performed using a multimode plate reader at a wavelength of 562 nm (EnSpire, Perkin Elmer, USA). All the results are plotted using Origin 2020 (Origin Lab, USA). All the schematics were sketched in Biorender.com.

Substrates (CNP-G3 & Starch) Size Measurement

Potato starch as large substrate and CNP-G3 (2-chloror-4-nitrophenyl alpha-D-maltotrisode) as small substrate were used as probe molecules for the activity estimation of nanoconfined porcine pancreatic α-amylase. Hydrodynamic size measurement were performed in Malvern zeta sizer nano ZS (UK). Both the substrates were solubilized in autoclaved, filtered (0.2 micron) MQ water (Merck Millipore, Germany). Starch was prepared at 5 mg/mL concentration and CNP-G3 at 4 mM concentration in MQ water.

Thermal Gravimetric Analysis (TGA) of α-amylase biosorbed silica

Thermal gravimetric analysis was performed on Silica – SBA-15 without α-amylase and a sample prepared with α-amylase loaded. For loading the α-amylase 1.5 µM of its solution (30mL) was incubated with 500 µg/mL silica dispersion. After incubated at 37 °C for 3 hours with vertical rotation (Harvard apparatus, cat no. 74-2302), silica was separated with by centrifugation. Both the samples with and without the α-

amylase were freeze dried, before being subjected to the TGA (Discovery, USA) at 2 °C/min. Obtained results from the analysis are plotted in Origin and presented in figure S8.

Post Synthesis Treatment of Particles

The MSP after calcination step was treated with acid in different solvents (toluene, ethanol, and water) after the synthesis and calcination step, following the general scheme 1. Each treatment was in a 20% v/v acidic solution. This is sketched in scheme 1 below.

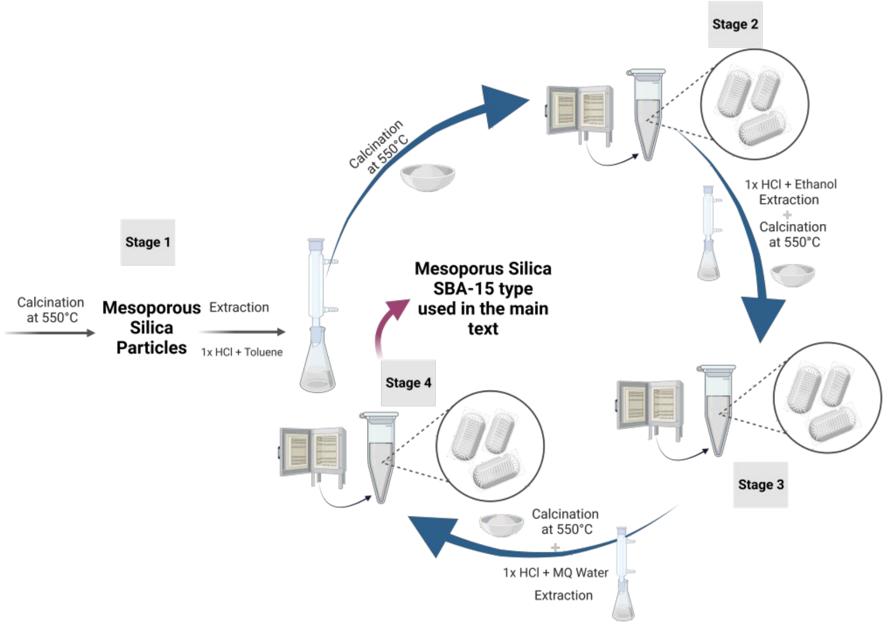

Scheme-1: Processing conditions and procedures for the different variants of the mesopores silica particles, used in this study. Results are reported in the main manuscript for only the MSP obtained after the third extraction that is stage 4 particles.

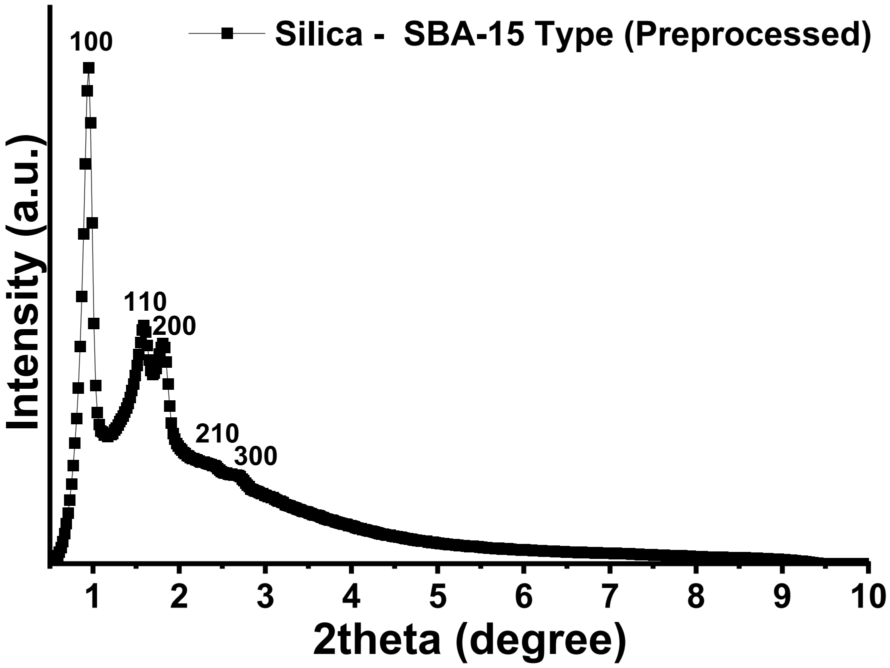

Figure S1: Low angle XRD of pre-ingested Nanoporous silica of SBA-15 at stage 1 from scheme SI-1.

Table S1: Biosorption analysis of MSPs after each stage of the treatment, values of parameters from nonlinear regression analysis using Hill equation.

Model	Hill			
Equation	$Q_e=m_{max}* C_e^n / (K^n+C_e^n)$			
Plot	Silica SBA-15 Type Stage 1	Silica SBA-15 Type Stage 2	Silica SBA-15 Type Stage 3	Silica SBA-15 Type Stage 4
m_{max} (μg/μg)	0,07 ± 0	0,22 ± 0	0,19 ± 0,01	0,21 ± 0,02
K (μM)	0,29 ± 0	0,32 ± 0	0,29 ± 0	0,25 ± 0.02
n	6,5 ± 0	7,43 ± 0.43	6,08 ± 0,75	3,58 ± 0,53
R^2	1,00	1,00	0,99	0,99

Table S2: MSP of SBA-15 type, obtained at different stages of treatment, and analysed as using gas sorption analysis.

Sample	BET	Total Pore Volume	Micropore t-plot	Average Pore Size Distribution (PSD)	FWHM from PSD
	(m^2/g)	(cm^3/g)	(m^2/g)	(nm)	(nm)
**Silica SBA-15 Type Stage 1 (Preprocessed)**	843±5	1.27±0.01	323±1	11.15±0.03	2.49±0.07
**Silica SBA-15 Type Stage 2**	520±10	1.02±0	125±5	11.02±0.03	2.38±0.07
**Silica SBA-15 Type Stage 3**	505±15	0.96±0	125±5	11.22±0,03	2.42±0.07
**Silica SBA-15 Type Stage 4 (Postprocessed)**	518±9	1.03±0	131±6	11.21±0.03	2.40±0.07

* Values in the table above are triplicate measurement and presented as mean±SD.

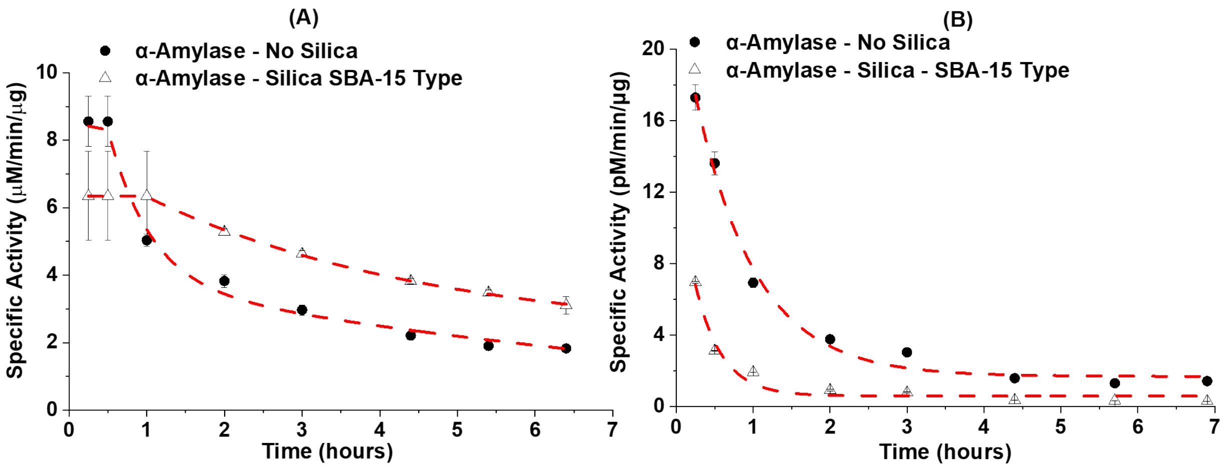

Figure S2: Time course of specific activity measurement in (A) using CNP-G3 (2mM) – small substrate and (B) with starch as substrate with loading amounts of substrate as 2mM and 3mg/mL respectively.

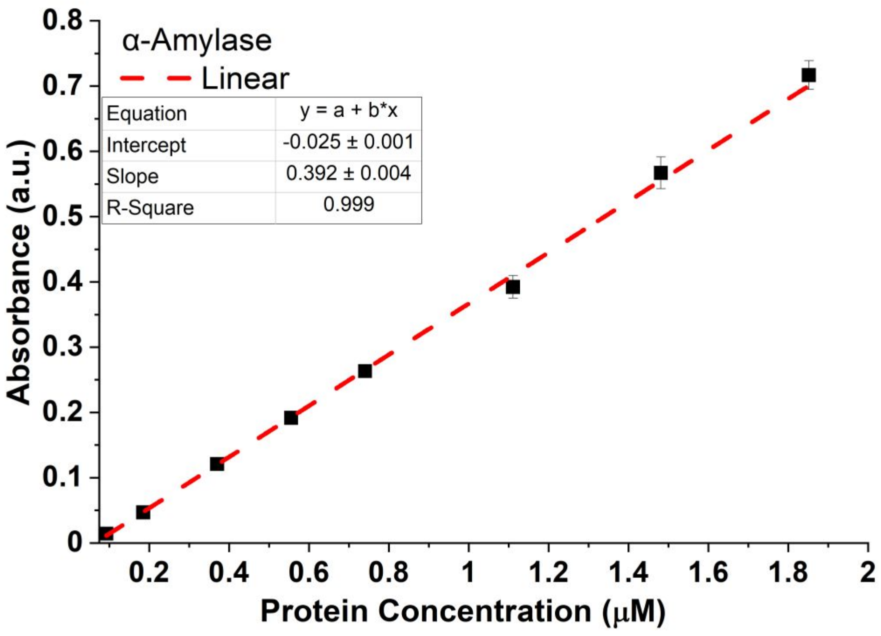

Figure S3: Standard curve for porcine pancreatic alpha-amylase measured at 562 nm OD values, in turn used for calculating the amount of α-amylase adsorbed in MSP, shown in adsorption plots in Figure 2. Dotted lines are linear fits. Mean of triplicated measurement is plotted with standard deviation presented in error bars.

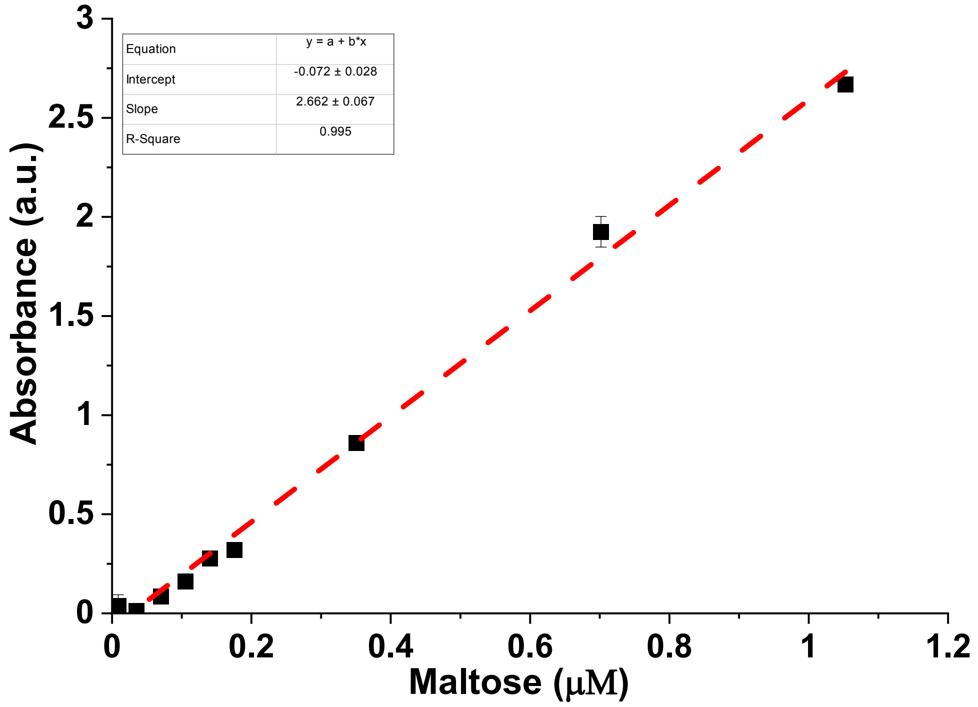

Figure S4: Standard curve for activity measurement of α-amylase using starch as a substrate with respect to dosing of α-amylase, which is presented in Figure S-1. Amount of reducing sugars produced was quantified using maltose as a standard curve.

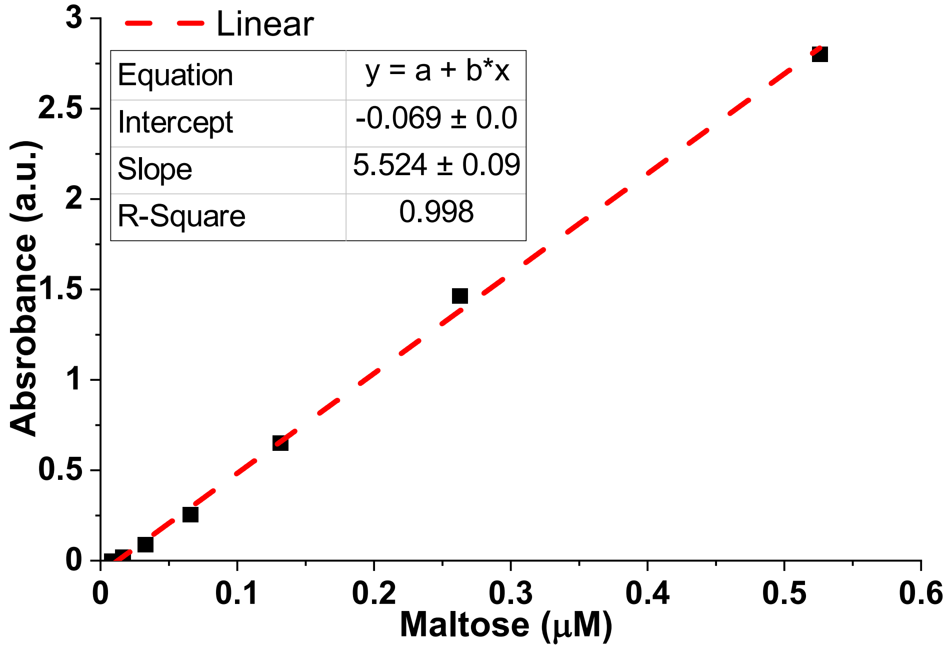

Figure S5: Standard Curve for activity measurement for α-amylase using large probe (starch) in figure 2 (B)

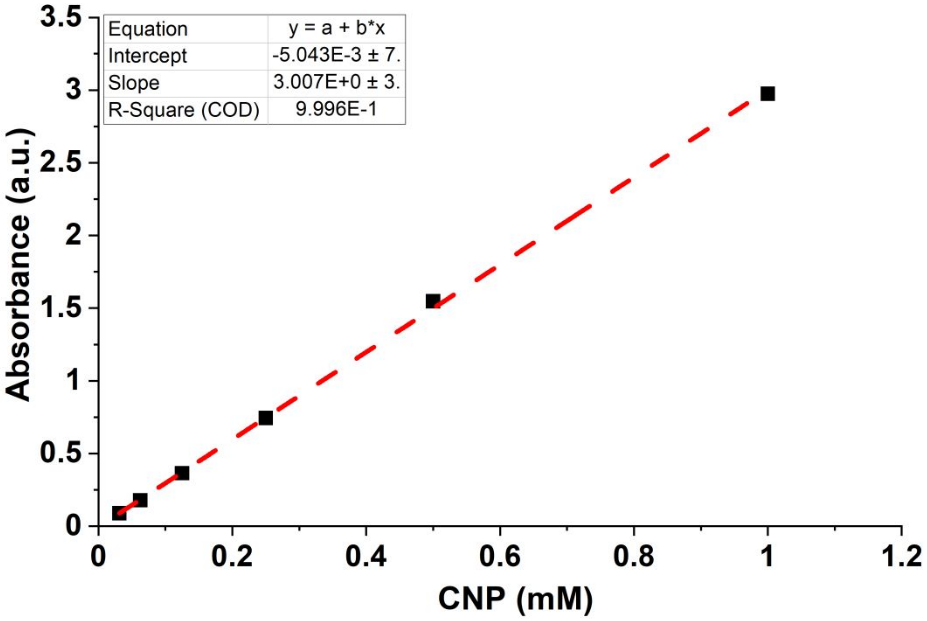

Figure S6: Standard curve for activity measurement of α-amylase using G3-CNP (structure in subsequent figure) as a substrate at a constant α-amylase concentration. Absorbance was recorded at 405 nm wavelength, and 2-cholor-4-nitrophenyl (CNP) was used a standard for quantification of amount of CNP cleaved.

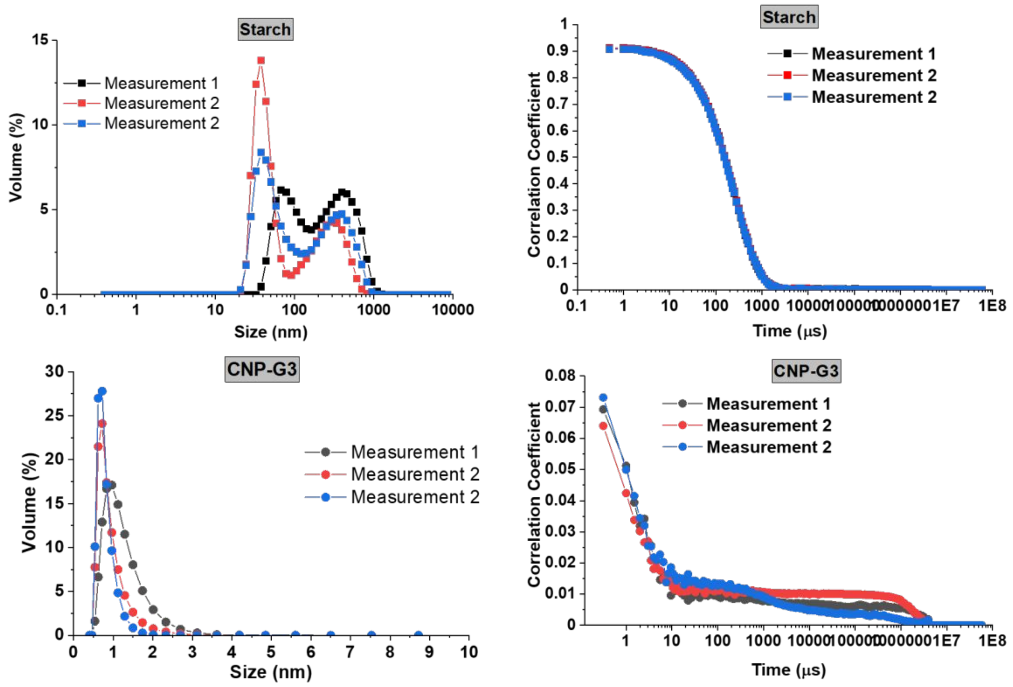

Figure S7: Starch and CNP-G3 substrates size estimation using zeta sizer. Left panel is the size distribution and right side panel is their corresponding correlation coefficients.

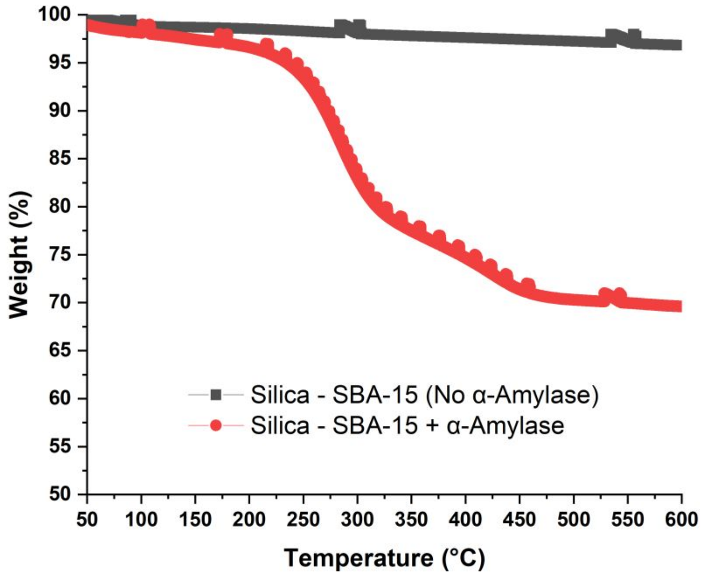

Figure S8: Thermal gravimetric analysis of α-amylase loaded silica particle (red curve) and without α-amylase.

References

[1] M. Thommes, K. Kaneko, A. V. Neimark, J. P. Olivier, F. Rodriguez-Reinoso, J. Rouquerol, K. S. W. Sing, *Pure Appl. Chem.* **2015**, *87*, 1051.
